# A Case of Pericardial Effusion in Association With Celiac Disease

**DOI:** 10.7759/cureus.67074

**Published:** 2024-08-17

**Authors:** Jaafar A Hamdan, Jahnavi Chaudhari, Thulfiqar Aljashamy, Safwan Mohiuddin, Denise Csendes, Nikolay Mitzov

**Affiliations:** 1 Internal Medicine, HCA Healthcare/University of South Florida (USF) Morsani College of Medicine/HCA Florida Oak Hill Hospital, Brooksville, USA

**Keywords:** cardiac tampon, gastric pathologies, cardiac pathologies, celiac disease, pericardial effusion

## Abstract

This is the case of a 22-year-old female with celiac disease-induced pericardial effusion. Celiac disease is a gastroenterological autoimmune condition that affects several organ systems. It is a disease found in both children and adults. As many systems are involved, this case presented with a unique presentation: pericardial effusion with symptoms overlapping those of cardiac chest pain such as substernal chest pain and shortness of breath. The patient had tachycardia, tachypnea, and jugular venous distention with distant S1 and S2 heart sounds. Cardiothoracic surgery was consulted and diagnosed the patient with pericardial effusion.

## Introduction

Pericardial effusion describes an acute or chronic fluid retention within the pericardial sac. The pericardial sac contains a potential space that is found between the visceral and parietal layers of the pericardium; this space contains 50 mL of serous fluid [1]. Any time the contents of that space exceed 50 mL, it becomes a pericardial effusion: a pathological picture. In this case, it is assessed with an objective factor, the pressure-volume relation (PVR). PVR conveys the clinical implication on the cardiac function in the setting of pericardial effusion [1]. As the pericardial effusion increases in volume, so does the PVR. The resulting high intrapericardial pressure leads to cardiac tamponade which can have a lethal outcome if not addressed in a timely manner [1]. There are many causes of pericardial effusion: idiopathic, infectious (e.g., viral, bacterial, fungal, protozoal), inflammatory (e.g., rheumatoid arthritis, connective tissue disease, vasculitis, medication-induced), post-myocardial infarction, hemopericardium, malignancies, and autoimmune conditions.

Pericardial effusion can be asymptomatic if the effusion is occurring chronically, where the cardiac muscles are able to adapt. However, in the setting of cardiac tamponade, which is often acute, the myocardium does not have time to adapt. Cardiac tamponade can present with hypotension, jugular venous distention (JVD), and muffled heart sounds, collectively termed "Beck's triad." Other features include dyspnea, tachypnea, and tachycardia [1]. On an electrocardiogram (EKG), this can be depicted as a reduction in voltage and electrical alternans. On chest radiography, you can appreciate a normal cardiac silhouette. However, if the pericardial effusion is moderate in size or larger, the cardiac silhouette will appear as a round flask-like shape [1]. At the bedside, a 2D transthoracic echocardiography (TTE) can be conducted to evaluate the presence of a pericardial effusion. This is a great tool to have when pericardiocentesis is warranted in order to relieve the PVR in the setting of significant pericardial effusion. When it comes to management, different avenues can be adopted; the main determinant to assess first however is determining if cardiac tamponade is present or if there are alarming features of tamponade. Some of those alarming features are suspected bacterial, fungal, or tuberculous pericarditis, intrapericardial hemorrhage, and moderate to large effusion that is increasing in size and is not chronic [1]. To give an example, large, asymptomatic, idiopathic effusions that persist three months or longer irrespective of therapy with anti-inflammatory regiments, for instance, in the setting of pericardial inflammation, will usually warrant pericardiocentesis [1]. Though pericardial effusion as mentioned has an extensive spectrum of etiologies/associations, a focus here will be placed on autoimmune pathologies, specifically celiac disease.

Celiac disease is a gastroenterological autoimmune condition which affects a broad span of organ systems. It is a disease found in both children and adults with a diagnosis mean age of 38 years old in the United States, overall affecting about 1% of the American population [2,3]. Ultimately, it is a condition resulting in enteropathy induced via gluten ingestion in those who are genetically susceptible (with anti-tissue transglutaminase antibodies) [2]. Gluten is a protein found in things like wheat, barley, and rye, which needless to say indicates a mainstay therapy of being on a gluten-free diet. However, persistent/recurrent symptoms can occur even in the setting of a gluten-free diet or even refractory form [2]. From a pathophysiological standpoint, in the gluten, there are gliadin peptides which lead to a series of events on the epithelial surface and the lamina propria via both the innate and the adaptive immune systems [2]. Some of the clinical features in those with celiac disease are short statures, failure to thrive during childhood, delayed onset of puberty, lethargy, weight loss, diarrhea, flatulence, and bloating/abdominal discomfort [2]. Though celiac disease involves extensive organ systems, a focus placed in this case will be on the cardiac system, specifically pericardial effusion in the background of celiac disease. In celiac disease, among the extra-intestinal manifestations of the disease, pericardial effusion is an unusual manifestation [3]. Per literature/case reports, pericardial effusion has been found incidentally, being non-symptomatic; interestingly enough, the pericardial effusion was being alleviated symptomatically with iron regimen supplements and a gluten-free diet [3].

## Case presentation

A 22-year-old female with a past medical history of autism and celiac disease presented with a chief complaint of shortness of breath associated with fever, chills, and substernal chest pain for one-week duration. The patient had moved from Cuba where she had limited access to healthcare. She was admitted to another facility prior and had computed tomography angiography (CTA) of the chest, revealing moderate to large non-hemorrhagic pericardial effusion. The patient was transferred to our facility for further management. Per the patient's family, she had a similar episode associated with upper respiratory symptoms in September 2023; however, the patient was still in Cuba where there was limited access to healthcare. On admission, her blood pressure was 114/77 mmHg, pulse 128, respiratory rate 32, oxygen saturation 98% on room air, and temperature 37.2°C. On physical exam findings, the patient appeared ill, and there were presence of right JVD, presence of bilateral carotid artery bruits, distant S1 and S2 heart sounds, and 1+ radial and posterior tibial pulses bilaterally. Table 1 below shows the abnormal lab findings of the patient during the initial encounter.

**Table 1:** Laboratory values on admission. This table shows the patient's lab values on admission to the hospital.

Parameters	Values	Reference values
Hemoglobin	9.1 g/dL	11.2-15.7 g/dL
Hematocrit	29.7%	34.1-44.9%
Mean corpuscular volume	68.4 fL	79.4-94.8 fL
Mean corpuscular hemoglobin	21.0 pg	25.6-32.2 pg
Mean corpuscular hemoglobin concentration	30.6 g/dL	32.2-35.5g/dL
Red cell distribution width	20.1%	11.7-14.4%
Platelet count	408 10^3^/uL	150-400 10^3^/uL
Sodium	134 mmol/L	136-145 mmol/L
Potassium	3.4 mmol/L	3.5-5.1 mmol/L
Blood urea nitrogen	3.0 mg/dL	7-18 mg/dL
Creatinine	0.5 mg/dL	0.6-1.0 mg/dL
Total bilirubin	1.5 mg/dL	0.2-1.0 mg/dL
Albumin	3.0 g/dL	3.4-5.0 g/dL

**Figure 1:**
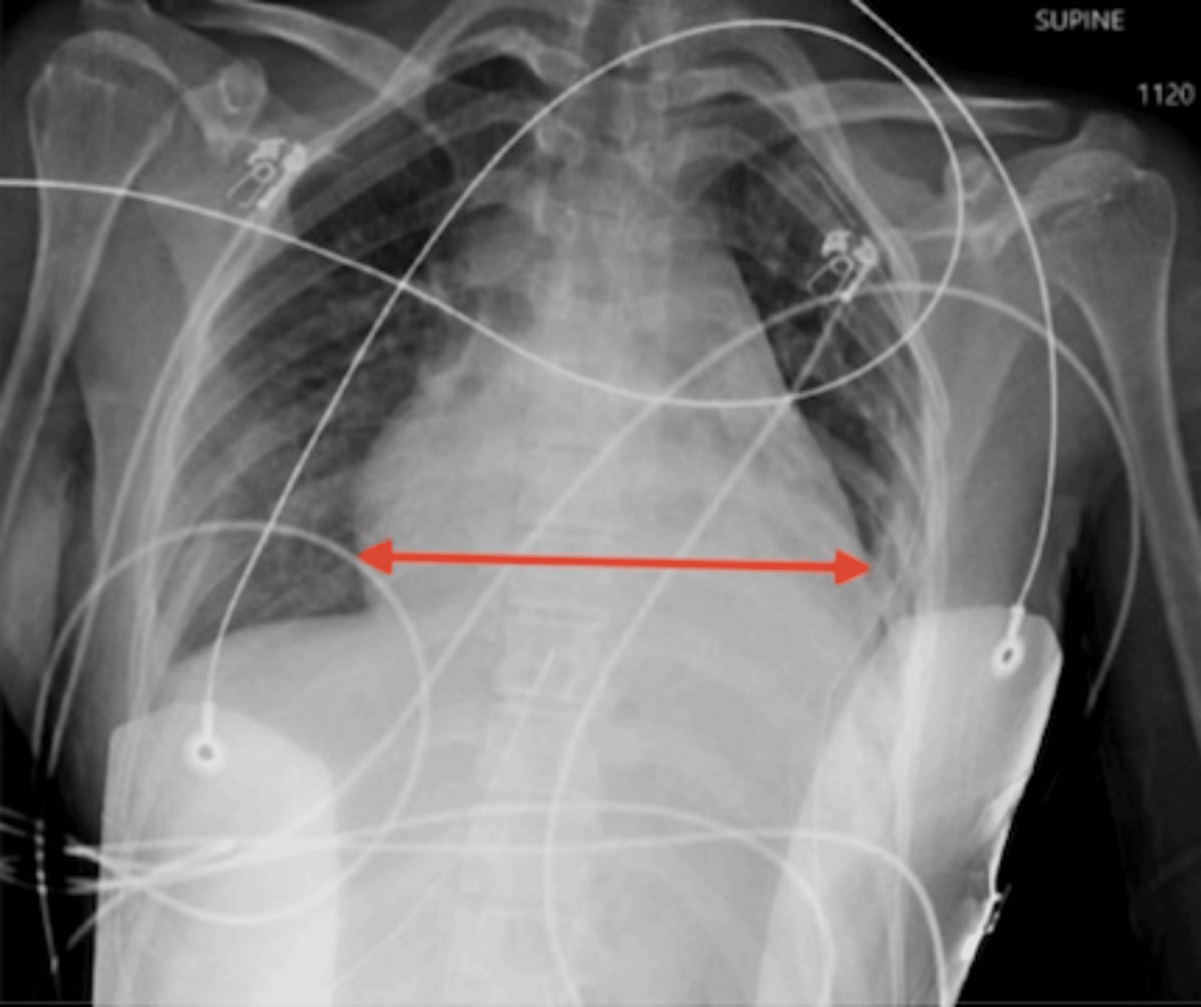
Chest X-ray. This is a depiction of a chest X-ray showing an enlarged cardiac silhouette consistent with pericardial effusion which is shown by the red arrow.

The patient's electrocardiograph showed sinus tachycardia, pulse 138, short PR interval, and no signs of active ischemia/infarction. On chest X-ray, there are enlarged cardiac silhouette consistent with known pericardial effusion and interval placement of left-sided thoracostomy tube with small residual left-sided pleural effusion as shown in Figure 1.

**Figure 2:**
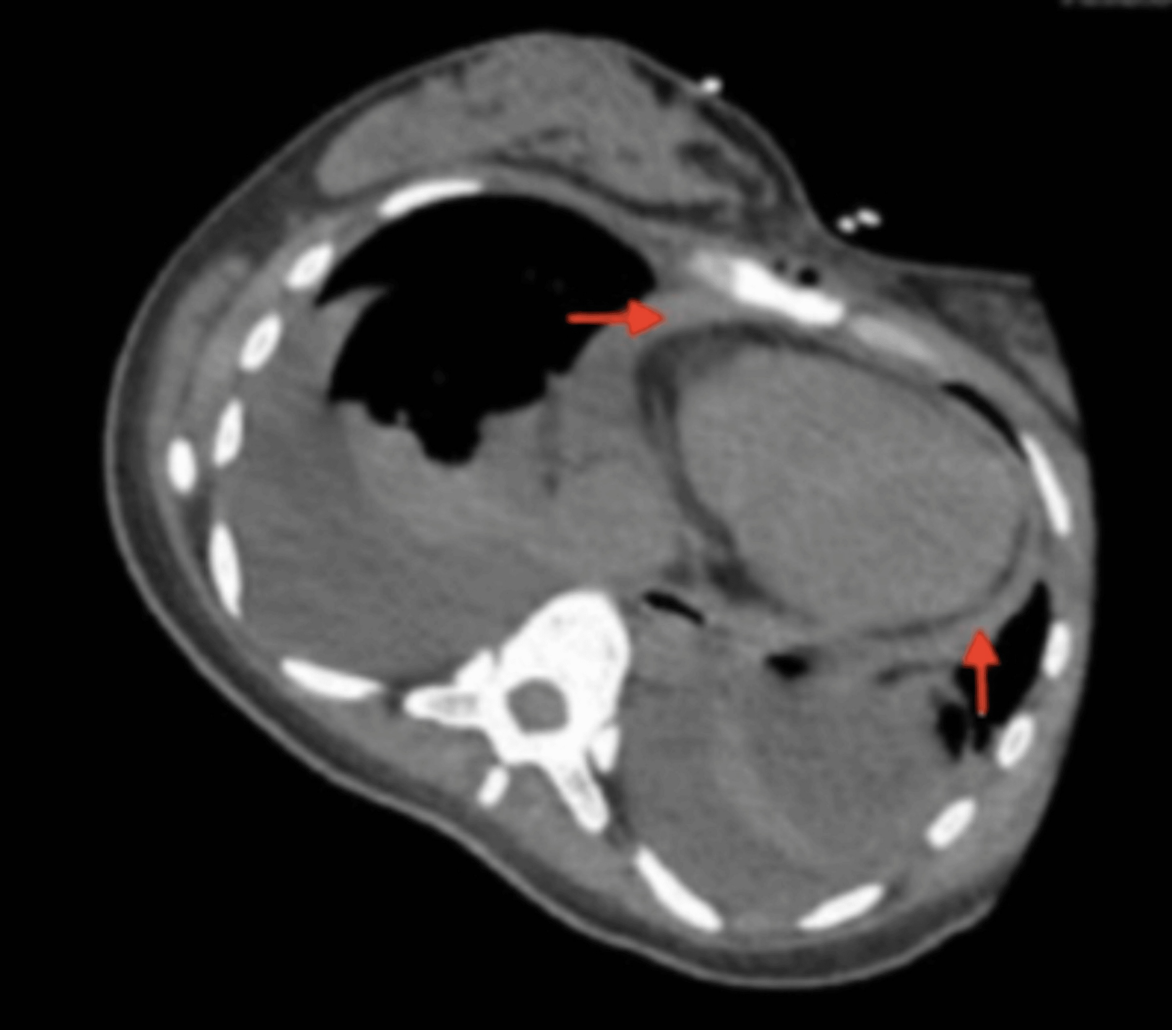
CTA. This is a CTA of the chest showing effusion within the pericardial sac. This is depicted by the red arrows in the image. CTA: computed tomography angiography

In subsequent studies, a CTA of the chest depicted a clearer image of the pericardial effusion that was present, as shown in Figure 2.

Cardiothoracic surgery was consulted; due to the image finding, the patient underwent a pericardial window, placement of pericardial tube and left chest tube with pericardial biopsy. On the echocardiogram, there was a pericardial effusion identified, with an estimated ejection fraction of 60%. Pericardial pleural fluid had scattered lymphocytes of mixed morphology, reactive mesothelial cells with few macrophages, and a small moderate amount of blood; there was no evidence of malignancy. On pericardial biopsy, there was fibrovascular and adipose connective tissue partly effaced by chronically inflamed granulation tissue; there was no evidence of necrotizing granuloma or malignancy. Blood cultures, pericardial fluid cultures, and gram stain were negative.

## Discussion

Pericardial effusion is an acute or chronic fluid retention within the pericardial sac. The pericardial sac has a space within, present between the visceral and parietal layers, which normally does contain up to 50 mL of serous fluid [1]. However, any additional to that rough amount of fluid normally present in that pericardial space is labeled pericardial effusion: a pathological picture our patient presented with. In this case, it is assessed with an objective factor, the PVR. PVR is a factor assessing the clinical implication on the cardiac function in the setting of pericardial effusion [1]. Normally, the PVR at its norm reflects a highly compliant pericardium, until the volume begins to increase above the norm, resulting in high intrapericardial pressure, ultimately resulting in cardiac tamponade which can have a lethal outcome if not addressed in a timely manner [1]. There are significant broad-spectrum causes of pericardial effusion etiologies: idiopathic, infectious (e.g., viral, bacterial, fungal, protozoal), inflammatory (e.g., rheumatoid arthritis, connective tissue disease, vasculitis, medication-induced), post-myocardial infarction, hemopericardium, malignancies, and autoimmune conditions. In our case, the patient had an autoimmune condition, celiac disease.

In the setting of pericardial effusion, some of the features seen are hypotension, JVD, and muffled heart sounds which are collectively termed Beck's triad which is what our patient was having. In addition, dyspnea, tachypnea, and tachycardia were seen in the patient upon presentation which are symptoms present in pericardial effusion as seen in literature [1]. Management of pericardial effusion, especially when it is acute, is pericardiocentesis to draw out the fluid and stabilize the patient hemodynamically. However, some of the underlying etiologies must be addressed/managed to prevent the recurrence of pericardial effusion. In the case of underlying comorbidity of celiac disease, though it is uncommon, it can still lead to pericardial effusion, particularly when iron deficiency is in the background; our patient had a mean corpuscular volume (MCV) of 68.4 fL. During hospitalization, hematology/oncology was consulted in which a recommendation was made for the patient to be given sodium ferric gluconate complex 125 mg intravenously twice daily for a total of eight doses. The patient was discharged on per os ferric citrate 210 mg daily, along with a pericardial window that had been created; the patient's symptoms and the pericardial effusion had resolved.

In celiac disease, among the extra-intestinal manifestations of the disease, pericardial effusion is an unusual manifestation [3]. Per literature/case reports, pericardial effusion has been found incidentally, being non-symptomatic; interestingly enough, the pericardial effusion was being alleviated with iron regimen supplements and a gluten-free diet [3]. Our patient was discharged on iron supplements and counseled extensively on the importance of a gluten-free diet and its positive implications. It is crucial, when one has pericardial effusion, to conduct a thorough history-taking, perform a physical examination, and investigate for any possible autoimmune conditions and lab abnormalities which could lead to this presentation.

## Conclusions

Celiac disease-induced pericardial effusion is an uncommon phenomenon which can be seen in the setting of iron deficiency due to the autoimmune condition, celiac disease. When one presents with abnormal vitals, where there is a concern for Beck's triad, it is important to conduct an emergent bedside echo to decide if pericardiocentesis needs to be done in addition to proper medical managements (e.g., replenishing iron stores).
